# Controlled Cycling and Quiescence Enables Efficient HDR in Engraftment-Enriched Adult Hematopoietic Stem and Progenitor Cells

**DOI:** 10.1016/j.celrep.2020.108093

**Published:** 2020-09-01

**Authors:** Jiyung J. Shin, Markus S. Schröder, Francisco Caiado, Stacia K. Wyman, Nicolas L. Bray, Matteo Bordi, Mark A. Dewitt, Jonathan T. Vu, Won-Tae Kim, Dirk Hockemeyer, Markus G. Manz, Jacob E. Corn

**Affiliations:** 1 Innovative Genomics Institute, University of California, Berkeley, CA 94720, USA; 2 Department of Molecular and Cell Biology, University of California, Berkeley, CA 94720, USA; 3 Department of Biology, ETH Zürich, 8093 Zürich, Switzerland; 4 Department of Medical Oncology and Hematology, University Hospital Zurich and University of Zurich, 8091 Zurich, Switzerland

**Keywords:** adult hematopoietic stem cells, gene editing, gene therapy, homology-directed repair, CRISPR, cell cycle, quiescence

## Abstract

Genome editing often takes the form of either error-prone sequence disruption by non-homologous end joining (NHEJ) or sequence replacement by homology-directed repair (HDR). Although NHEJ is generally effective, HDR is often difficult in primary cells. Here, we use a combination of immunophenotyping, next-generation sequencing, and single-cell RNA sequencing to investigate and reprogram genome editing outcomes in subpopulations of adult hematopoietic stem and progenitor cells. We find that although quiescent stem-enriched cells mostly use NHEJ, non-quiescent cells with the same immunophenotype use both NHEJ and HDR. Inducing quiescence before editing results in a loss of HDR in all cell subtypes. We develop a strategy of controlled cycling and quiescence that yields a 6-fold increase in the HDR/NHEJ ratio in quiescent stem cells *ex vivo* and *in vivo*. Our results highlight the tension between editing and cellular physiology and suggest strategies to manipulate quiescent cells for research and therapeutic genome editing.

## Highlights

• HSCs require more time to activate HDR pathways than do progenitor cells
• Rapamycin and CHIR99021 in media induce quiescence and maintain stemness in HSPCs
• Quiescent HSPCs perform only NHEJ, whereas cycling HSPCs perform both NHEJ and HDR
• Inducing quiescence after a short period of cycling yields HDR in engrafting HSCs

## Graphical Abstract

**Figure:**
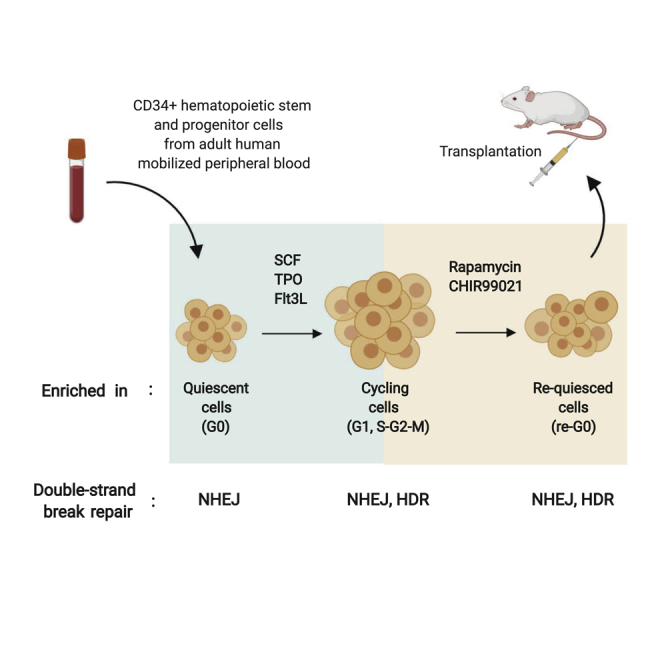

## Teaser

Shin et al. find that quiescent blood stem cells only perform error-prone NHEJ to repair double-strand breaks, whereas cycling blood stem cells perform both error-prone NHEJ and precise HDR. They show that inducing quiescence after a short period of cycling yields blood stem cells harboring high levels of HDR.

## Introduction

CRISPR-Cas genome editing has emerged as a powerful tool that enables fundamental research into genotype-phenotype relationships and holds great promise for the treatment of genetic disease (Doudna and Charpentier, 2014; Fellmann et al., 2017; Sternberg and Doudna, 2015). Double-stranded DNA damage induced by CRISPR-Cas enzymes can be repaired by either error-prone non-homologous end joining (NHEJ) to create indels and disrupt a locus or templated homology-directed repair (HDR) to precisely change a sequence. Cell cycle plays an important role in DNA repair decisions in response to double-strand breaks (DSBs). In most human cell types, NHEJ is the primary repair mechanism throughout the cell cycle, whereas HDR occurs at a much lower rate and primarily happens in S/G2 phase due to template availability and to avoid inappropriate telomere fusion during mitosis (Branzei and Foiani, 2008; Essers et al., 2002; Hustedt and Durocher, 2016; Mao et al., 2008; Orthwein et al., 2014, 2015; Pietras et al., 2011; Saleh-Gohari and Helleday, 2004). The high levels of NHEJ and correspondingly low levels of HDR in primary cells have complicated both fundamental research and therapeutic applications that make use of genome editing.

**Figure 1:**
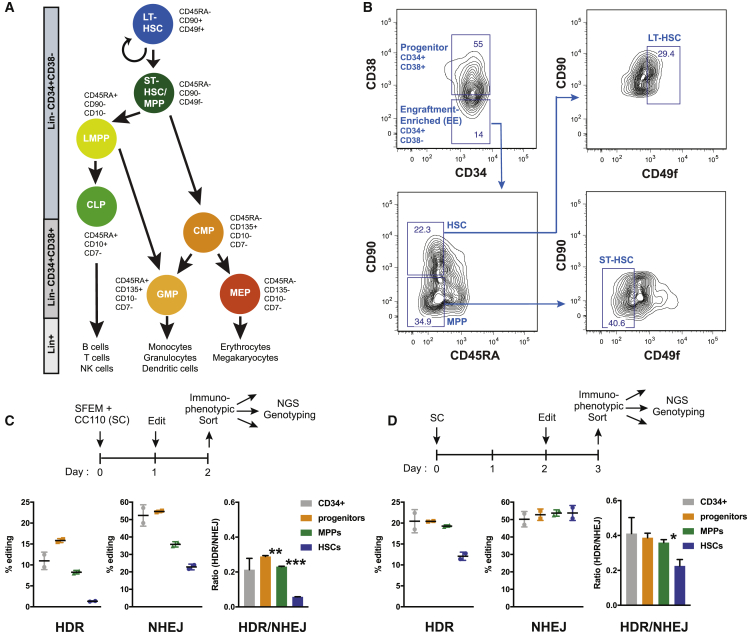
HSCs Require More Time to Activate HDR Pathways during Gene Editing Than Differentiated Cells (A) Diagram describing the human hematopoietic population hierarchy: long-term hematopoietic stem cell (LT-HSC), short-term hematopoietic stem cell (ST-HSC), multipotent progenitor (MPP), lymphomyeloid-primed progenitor (LMPP), common lymphoid progenitor (CLP), common myeloid progenitor (CMP), granulocyte monocyte progenitor (GMP), and megakaryocytic-erythroid progenitor (MEP). Immunophenotypic markers for each subpopulation were adapted from (Corces et al., 2016; Notta et al., 2011). (B) Fluorescence-activated cell sorting scheme to isolate human progenitors (CD34+ CD38+), engraftment-enriched (EE) HSPCs (CD34+ CD38−), MPPs, HSCs, ST-HSCs, and LT-HSCs from human mobilized peripheral blood (mPB) CD34+ HSPCs. CD34+ cells were stained with monoclonal antibodies against CD34, CD38, CD45RA, CD90 (Thy1), and CD49f antigens. The frequency of each subpopulation is based on the parent gate. (C) Editing outcomes in CD34+ subpopulations 1 day post-electroporation and 2 days in culture. Percentage of reads positive for HDR or NHEJ by next-generation amplicon sequencing at the HBB site. HSCs lack HDR alleles. Representative data from experiments performed with three different mPB donors and n = 3 biological replicates per donor. Mean ± SD shown. ^∗∗^p < 0.01, ^∗∗∗^p < 0.001 by unpaired t test. (D) Editing outcomes in CD34+ compartments 1 day post-electroporation and 3 days in culture. Percentage of reads positive for HDR or NHEJ by next-generation amplicon sequencing at the HBB site. HSCs accumulate significant HDR alleles, although they show a lower HDR/NHEJ ratio than that of MPPs and progenitors. Representative data from experiments performed with three different mPB donors and n = 3 biological replicates per donor. Mean ± SD shown. ^∗∗^p < 0.05 by unpaired t test. See also Figure S1.

Primary hematopoietic stem cells (HSCs) ensure the lifelong production of all blood cells through their unique capacity to self-renew and to differentiate (Figure 1A). Inadequate HSC renewal can lead to severe anemia, such as Fanconi anemia and Diamond-Blackfan anemia (Corey et al., 2007). Inappropriate differentiation can lead to either the over- or under-production of blood components, causing disorders that range from immunodeficiency to cancer.

Due to their ability to simultaneously self-renew and generate the entire blood system, long-term HSCs (LT-HSCs) represent an attractive target for genome editing to investigate the mechanisms of inherited blood disorders and to deliver lasting treatments. CRISPR-Cas genome editing has emerged as an effective tool to precisely target human HSCs, but the replacement of genetic sequences by nuclease-induced HDR in HSCs has lagged behind the ability to disrupt sequences by NHEJ in these cells (Dever et al., 2016; DeWitt et al., 2016; Genovese et al., 2014b; Hoban et al., 2015; De Ravin et al., 2017; Wang et al., 2015). Although bulk-edited CD34+ populations of hematopoietic stem and progenitor cells (HSPCs) exhibit high levels of HDR after a few days in culture, it has been highly challenging to maintain long-term engraftment of HDR-edited HSCs (Dever et al., 2016; DeWitt et al., 2016; Genovese et al., 2014b; Hoban et al., 2015; Wang et al., 2015). Without introducing a selectable marker to isolate HDR events, the prevalence of HDR alleles in LT-HSCs ranges from less than 1% to ∼2.5% (DeWitt et al., 2016; Genovese et al., 2014b; Hoban et al., 2015; Wang et al., 2015). The paucity of nuclease-induced HDR in LT-HSCs could stem from a number of factors, including inefficient delivery of the HDR donor used to program the change, cell toxicity introduced by the act of performing HDR itself, or fundamental limitations on HDR imposed by the underlying biology of LT-HSCs.

HSCs, like many other adult stem cells, can exist in both cycling and G0 quiescent states. For HSCs, cycling supports hematopoiesis, whereas quiescence preserves the stem cell population (Li and Clevers, 2010). Quiescent HSCs from mice primarily use NHEJ in response to non-specific DSBs, whereas cycling mouse HSCs can use both NHEJ and HDR (Beerman et al., 2014; Mohrin et al., 2010). But human HSCs are distinct from their mouse counterparts in terms of the frequency of cycling (Abkowitz et al., 1996; Cheshier et al., 2007; Kiel et al., 2007; Nombela-Arrieta and Manz, 2017), DNA damage response (Biechonski and Milyavsky, 2013; Mohrin et al., 2010), and expression of DSB repair genes (Biechonski and Milyavsky, 2013). The use of cell-cycle-regulated Cas9 constructs in human HSPCs has enabled decreases in deleterious NHEJ alleles, thereby improving HDR/NHEJ ratios (Lomova et al., 2018). But, explicitly increasing HDR alleles in quiescent LT-HSCs has proven elusive.

Here, we investigate the relationship between the cell cycle status of adult human mobilized peripheral blood (mPB) CD34+ HSPC subpopulations and their editing outcomes. We find that editing CD34+ HSPCs results in high levels of HDR in relatively differentiated subpopulations, but G0 HSPCs almost completely lack HDR alleles. Allowing HSPCs to briefly enter the cell cycle yields immunophenotypically primitive cells (CD34+ CD38−) with high levels of HDR but few quiescent cells. We define these CD34+ CD38− immunophenotypically primitive cells as “engraftment-enriched” (EE) HSPCs for the purpose of this paper because CD34+ CD38− HSPCs have been shown to primarily consist of cells that preserve the potential to engraft (Bhatia et al., 1997; Hao et al., 1995; Hogan et al., 2002; Zonari et al., 2017). Although CD90, EPCR, or ITGA3 more efficiently enriches for primitive cells in cultured HSPCs than in CD38 (Martin and Park, 2017; Tomellini et al., 2019), we used CD38 becauase antibodies for markers other than CD38 that we tested were not compatible with the fixation step for the simultaneous cell cycle analysis. Using the timed administration of a small molecule cocktail originally developed for HSC maintenance, we developed a protocol to place HDR-edited EE HSPCs back into quiescence. The end result is G0 EE HSPCs whose HDR editing efficiency reflects the rest of the CD34+ HSPC population. This finding translates to an almost 6-fold increase in HDR/NHEJ ratios of EE HSPCs. Similar increases in HDR/NHEJ ratios were found during xenotransplantation *in vivo*, confirming that the re-quiescence protocol leads to higher levels of HDR in cells with long-term stem cell potential. These data yield insights into the DNA repair preferences of HSPCs enriched for engrafting cells and suggest routes to therapeutic protocols for efficient genome editing to cure blood disorders.

## Results

### HSCs Require More Time to Activate HDR Pathways during Gene Editing Than Differentiated Cells Require

Although gene editing reagents have been used to induce significant levels of HDR editing in bulk CD34+ HSPCs, the maintenance of HDR for a prolonged period of time after *in vivo* engraftment has been challenging (Dever et al., 2016; DeWitt et al., 2016; Genovese et al., 2014b; Hoban et al., 2015; Wang et al., 2015). In contrast, NHEJ is maintained at high levels during prolonged engraftment. This could either arise because the act of editing somehow makes LT-HSCs lose markers of stemness or because LT-HSCs do not perform HDR. To address this dichotomy, we first interrogated the extent to which primitiveness affects the repair decision after a Cas9-induced DSB in human mPB CD34+ HSPCs.

We used a potent single guide RNA (sgRNA) we previously found to efficiently edit human CD34+ HSPCs at the hemoglobin beta (HBB) locus and an single-stranded oligodeoxynucleotides (ssODN) donor template designed to modify the causative *HBB* mutation involved in sickle cell disease (SCD) (Figure S1A; Cradick et al., 2013; DeWitt et al., 2016). After editing bulk CD34+ HSPCs, we measured the efficiency of HDR and NHEJ in immunophenotypically sorted HSCs (CD34+ CD38− CD45RA− CD90+), multipotent progenitors (MPPs; CD34+ CD38− CD45RA− CD90−), and progenitors (CD34+ CD38+) (Figures 1A and 1B). Editing efficiency was quantified by using next-generation amplicon sequencing encompassing the HBB target site (Figure S1B).

We cultured CD34+ HSPCs in stem cell expansion media consisting of SFEMII and CC110 cytokine cocktail (SC) for 1 day, electroporated the cells with HBB-targeting Cas9 ribonucleoprotein complexes (RNPs), and cultured the HSPCs for 1 day before separating several HSPC subsets by using fluorescence-activated cell sorting (FACS) and assessing the editing efficiency in each subset through next-generation sequencing (NGS) genotyping (Figure 1C, top). Both HDR and NHEJ were evident in bulk CD34+ cells and relatively differentiated progenitors (CD34+ CD38+). Total editing was somewhat reduced in MPPs (CD34+ CD38− CD45RA− CD90−). Strikingly, we found moderate amounts of NHEJ in immunophenotypic HSCs (CD34+ CD38− CD45RA− CD90+) but almost no HDR in these cells, which led to a 3-fold lower HDR/NHEJ ratio in HSCs than to bulk CD34+ HSPCs. (Figure 1C).

We further cultured the sorted populations (HSCs, MPPs, and progenitors) and found that HSCs eventually accumulated HDR edits but only 72 h after electroporation (Figure S1C). However, the HDR/NHEJ ratio was highest in progenitors and lowest in HSCs even 72 h after electroporation (Figure S1C). In contrast, keeping CD34+ HSPCs in culture for 2 days before electroporation led to the appearance of significant HDR edits just 1 day after electroporation (Figure 1D). HDR was evident in all HSPC subtypes, including HSCs. These data indicate that more primitive HSCs preferentially repair Cas9-induced DSBs by NHEJ, but additional time in culture before the introduction of a DSB activates pathways related to HDR.

### Establishing the Timing of Cell Cycle Status in CD34+ Subsets during *Ex Vivo* Culture

HSPC primitiveness is linked to slower entry into the cell cycle (Laurenti et al., 2015) as well as lower frequency of cell cycle (Bradford et al., 1997; Morrison and Weissman, 1994; Pietrzyk et al., 1985; Suda et al., 1983; Uchida et al., 2003), and cell cycle progression is a major hallmark of increasing time in culture for HSPCs. Because HDR is intimately linked with cell cycle, we hypothesized that HSCs cannot use HDR at short culture time points due to quiescence resulting from slow entry into the cell cycle.

**Figure 2:**
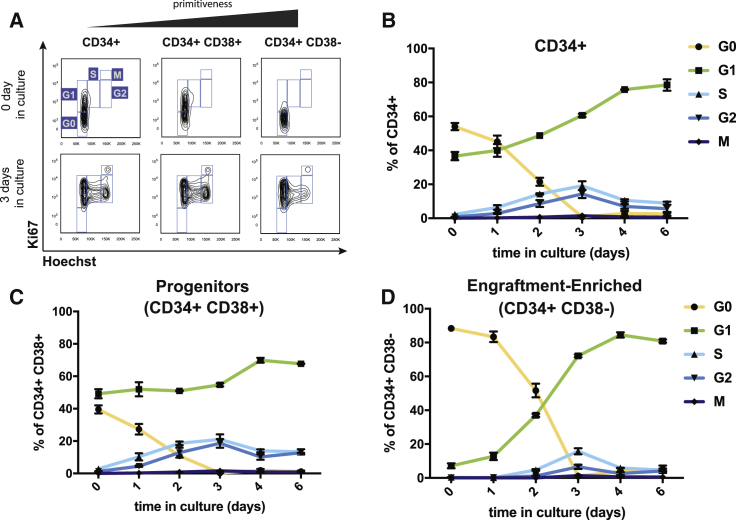
Cell Cycle Progression of Human mPB CD34+ Cells in *Ex Vivo* Culture (A) Representative flow cytometry plots for assessing cell cycle status in CD34+, CD34+ CD38+ (progenitors), and CD34+ CD38− (EE) populations. CD34+ cells were stained with antibodies against Ki67 and Hoechst 33342. G0: 2N DNA and Ki67 negative, G1: 2N DNA and Ki67 positive, S/G2/M: 4N DNA and Ki67 positive. (B) Cell cycle status of CD34+ cells in *ex vivo* culture. Notably, ∼60% of bulk CD34+ cells are in G0 at day 0, and 100% of the cells are cycling by day 3. (C) Cell cycle status of CD34+38+ progenitor cells in *ex vivo* culture. More than 50% of progenitor cells are cycling at day 0, and the percentage of cycling cells continually increase until day 3. (D) Cell cycle status of CD34+38− EE HSPCs in *ex vivo* culture. Notably, ∼90% of the EE HSPCs are quiescent at day 0, and EE HSPCs gradually exit quiescence until day 3 where 100% of them are cycling. Representative data from experiments performed with three different mPB donors and n = 2 biological replicates per donor. See also Figure S2.

Although the cycling properties of freshly isolated mouse and human HSC subpopulations have been described (Benveniste et al., 2010; Cheshier et al., 1999; Copley et al., 2012; Foudi et al., 2009; Laurenti et al., 2015; Oguro et al., 2013; Passegué et al., 2005; Qiu et al., 2014; Wilson et al., 2008), the cycling properties of human CD34+ HSPCs during extended *ex vivo* culture are not fully established. Before investigating the relationship between cell cycle status and editing efficiency, we first explored the cell cycle progression of CD34+ cells in *ex vivo* culture by using immunophenotyping combined with Hoechst 33342 (stains for DNA) and Ki67 (highly expressed in proliferating cells) staining. (Gerdes et al., 1984; Kim and Sederstrom, 2015). We found that more than 50% of cryopreserved mPB CD34+ HSPCs are quiescent (in G0) when thawed, but they gradually enter the cell cycle and are fully cycling by 3 days in SC culture (Figures 2A, 2B, and S2).

We next examined CD34+ CD38− HSPCs, which contain most of the engraftment potential within the CD34+ population and are enriched for primitive populations, such as HSCs and MPPs, as well as the more differentiated progenitor CD34+ CD38+ populations. For simplicity, here, we define CD34+ CD38− HSPCs as EE HSPCs. Interestingly, EE HSPCs have a delayed exit from quiescence compared to progenitors (Figures 2A, 2C, 2D, and S2). When cells are edited after only 1 day in culture (Figure 1C), 80% of EE HSPCs are quiescent at the time of editing, whereas 70% of CD34+ CD38+ progenitors are cycling (Figures 2C, 2D, and S2). These results support the absence of HDR in quiescent cells and correlate with the vast difference in HDR efficiency between HSCs and progenitors (Figure 1C). In contrast, when cells are edited after 2 days in culture (Figure 1D), more than 50% of the EE HSPCs have begun to actively cycle. This finding could account for the significant amount of HDR observed in HSCs during longer *ex vivo* culture (Figures 1D, 2C, and 2D).

### Quiescent CD34+ HSPCs Perform Only NHEJ, but Cycling CD34+ HSPCs Perform both NHEJ and HDR

**Figure 3:**
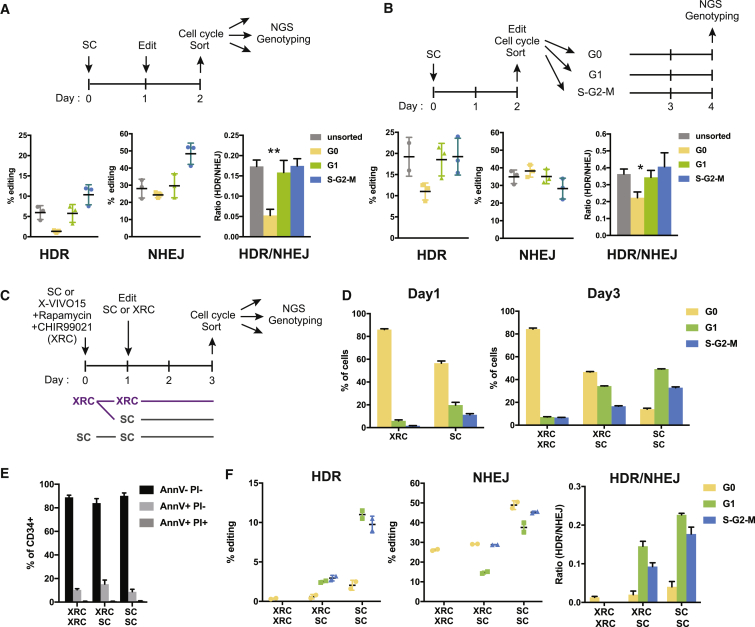
Quiescent CD34+ HSPCs Perform Only NHEJ, but Cycling CD34+ HSPCs Perform Both NHEJ and HDR (A) Editing outcomes in CD34+ subpopulations in different cell cycle status. One day post-electroporation and 2 days in culture. Percentage of reads positive for HDR or NHEJ by next-generation amplicon sequencing at the HBB site. G0 CD34+ HSPCs in 2-day culture do not accumulate HDR alleles. Representative data from experiments performed with three different mPB donors and n = 3 biological replicates per donor. Mean ± SD shown. ^∗∗^p < 0.01 by unpaired t test. (B) Editing outcomes in CD34+ subpopulations sorted into different cell cycle statuses. Two days post-electroporation and 4 days in culture. Percentage of reads positive for HDR or NHEJ by next-generation amplicon sequencing at the HBB site. G0 CD34+ HSPCs sorted at 2 days in culture accumulate significant HDR alleles in 4-day culture. Representative data from experiments performed with three different mPB donors and n = 3 biological replicates per donor. Mean ± SD shown. ^∗^p < 0.05 by unpaired t test. (C) Schematic of the workflow for mTOR and GSK-3 inhibition with rapamycin and CHIR99021 (XRC) for inhibition of cell cycle entry. Culture condition are SFEMII + CC110 (SC) and X-VIVO15 + rapamycin + CHIR99021 (XRC). (D) Cell cycle profiles of CD34+ cells in SC or XRC at the time of electroporation (day 1) and two days post-nucleofection (day 3). XRC prevents cell cycle entry, but this can be reversed by placing the CD34+ cells in SC media. Representative data from experiments performed with three different mPB donors and n = 2 biological replicates per donor. Mean ± SD shown. (E) Percentage of early and late apoptosis was assessed by staining the cells for annexin V (AnnV) and propidium iodide (PI) at 2 days post-nucleofection. AnnV−PI−, viable; AnnV+PI−, early apoptotic; AnnV+PI+, apoptotic. XRC does not induce apoptosis. Representative data from experiments performed with three different mPB donors and n = 2 biological replicates per donor. Mean ± SD shown. (F) Editing outcomes in CD34+ cells kept in SC media or XRC in different cell cycle status 2 days post-nucleofection and 3 days in culture. Inhibition of cell cycle entry by XRC blocks HDR repair but is reversible. Representative data from experiments performed with three different mPB donors and n = 2 biological replicates per donor. Mean ± SD shown. See also Figure S3.

To directly test how cell cycle status affects editing of human adult stem cells, we edited CD34+ HSPCs after 1 day in culture, allowed them to resolve edits for another day in culture, sorted them by cell cycle status, and used amplicon NGS to assess each population’s editing outcomes (Figures 3A, top, and S3A). One day after editing, we found that cells in G1 and S-G2-M stages had a substantial amount of HDR alleles, but quiescent G0 CD34+ HSPCs almost completely lacked HDR alleles and had a 3-fold decrease in the HDR/NHEJ ratio compared to cycling HSPCs (Figure 3A). We observed NHEJ alleles in significant amounts regardless of cell cycle, although the highest amount was observed in the S-G2-M population (Figure 3A). Intriguingly, 6 h after editing, we found small amounts of NHEJ alleles across various cell cycle subpopulations, but HDR alleles do not appear in any of the cell cycle subpopulations, which is consistent with reports from other cell types that found HDR takes longer than NHEJ (Arnoult et al., 2017; Mao et al., 2008; Figure S3B).

We next asked whether additional time in culture altered CD34+ HSPC editing outcomes according to cell cycle status. Because Hoechst staining led to a significant decrease in viability in CD34+ HSPCs (Figures S3C and S3D), we developed a live cell staining protocol that uses Pyronin Y that can stain both DNA and RNA when used alone (Darzynkiewicz et al., 1987, 2004). Cells were cultured for 2 days and then edited and immediately subjected to a live cell cycle sort using Pyronin Y accumulation (Figures 3B, top, and S3E). Sorted subpopulations were cultured for an additional 2 days before NGS genotyping to allow edits to resolve according to cell cycle status (Figure 3B, top). Similar to short-culture experiments, we observed NHEJ in cells regardless of cell cycle (Figure 3B, middle). Unlike short-culture experiments, quiescent G0 cells kept in culture for a total of 4 days displayed substantial HDR alleles (Figure 3B, left).

Because almost all CD34+ cells exit quiescence within 3 days in culture (Figure 2), CD34+ HSPCs that are still in G0 at the time of editing (mostly CD34+ CD38− EE HSPCs) should exit quiescence by the end of a long-term culture and would be able to accumulate significant HDR alleles while cycling. Hence, our results overall suggest that non-cycling CD34+ HSPCs in G0 are highly enriched in primitive EE HSPCs and heavily rely on the NHEJ pathway, as opposed to HDR. In contrast, cycling cells in G1 and S-G2-M are enriched in more differentiated CD34+ CD38+ progenitors and use both HDR and NHEJ.

### Preventing Exit from Quiescence Blocks HDR Repair in CD34+ HSPCs

Our previous experiments showed that quiescent, primitive HSPC subsets are less likely to perform HDR than cycling, differentiated subsets. We next tested whether induction of quiescence was sufficient to affect HDR levels under otherwise HDR-competent conditions, thereby directly testing whether quiescence was the causative factor of reduced HDR in HSCs.

We induced quiescence by using either retinoic acid, which has been shown to drive mouse HSCs into deep dormancy (Cabezas-Wallscheid et al., 2017), or inhibitors of mammalian target of rapamycin (mTOR) (rapamycin) and GSK-3 (CHIR9901), which have been used to maintain mouse and human HSCs *ex vivo* and *in vivo* (Huang et al., 2012; Figure S3F). We found that treatment of CD34+ HSPCs with retinoic acid in SC media led to differentiation, as measured by the substantial loss of CD34 expression, which could potentially be due to the differences in the maintenance of HSCs in mouse and human, whereas a combination of rapamycin and CHIR99021 in X-VIVO media (XRC) led to the prevention of cell cycle entry while maintaining primitiveness (Figures S3F–S3H).

We investigated editing outcomes in CD34+ HSPCs cultured in XRC as compared to SC expansion media. We used three different treatment regimens (Figure 3C). One set of HSPCs was kept in SC both before and after editing. A second set was started in XRC before editing and then either maintained in XRC after editing or moved to SC after editing. All cells were sorted based on cell cycle, and editing outcomes for each stage of the cell cycle were measured by NGS. Cells maintained in SC media entered cell cycle as normal, exhibiting a decrease in G0 cells and increase in G1 and S-G2-M cells after 3 days. Pre-treatment of CD34+ HSPCs with XRC led to the prevention of cell cycle entry, with almost all cells in G0 after 3 days (Figure 3D). Treatment with XRC was not associated with a decrease in cell viability (Figure 3E), and moving XRC-treated cells to SC media allowed HSPCs to re-enter the cell cycle, as measured by a decrease in G0 cells and increase in G1 and S-G2-M (Figure 3D).

Strikingly, quiescent CD34+ HSPCs treated continuously with XRC repaired almost all Cas9-induced DSBs by using NHEJ and harbored almost undetectable levels of HDR alleles (Figure 3F). Moving XRC-treated HSPCs to SC media after editing led to increased levels of HDR, but this result was mostly confined to cells in G1 and S-G2-M. HSPCs maintained in SC before and after editing exhibited low levels of HDR in G0 cells, but high levels of HDR in G1 and S-G2-M (Figure 3F). These results show that small-molecule-induced quiescence in HSPCs is sufficient to prevent HDR even after multiple days in *ex vivo* culture and that cycling is necessary for high levels of HDR.

### Inducing Quiescence after a Short Period of Cycling Yields Quiescent, Primitive HSPCs That Harbor HDR Alleles

Although XRC treatment has previously been used to maintain stemness (Huang et al., 2012), we next asked whether these compounds could induce quiescence after HSPCs have been allowed to cycle. Our overall goal was to allow HSPCs to cycle to accumulate HDR alleles during editing and then to place them back into G0 to maintain stemness.

**Figure 4:**
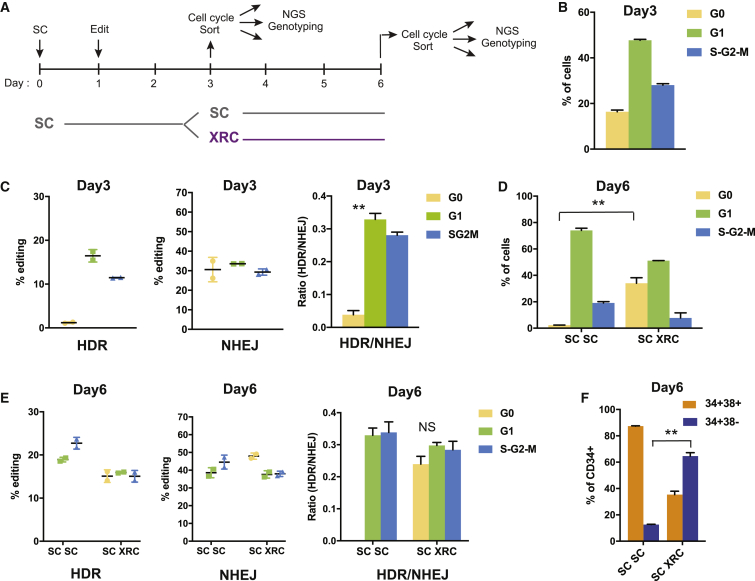
Inducing Quiescence after a Short Period of Cycling Yields Quiescent, Primitive HSPCs That Harbor HDR Alleles (A) Schematic of the workflow for inducing quiescence after a short period of cycling. CD34+ HSPCs are placed in SC culture for 1 day before editing and cycle for 2 additional days in SC culture, and then quiescence is induced with XRC for 3 days before the cells are subjected to FACS based on their cell cycle status and genotyped by NGS. (B) Cell cycle profiles of CD34+ cells 3 days in culture (SC). Most CD34+ HSPCs are cycling at day 3. Representative data from experiments performed with three different mPB donors and n = 2 biological replicates per donor. Mean ± SD shown. (C) Editing outcomes in CD34+ cells 3 days in culture (SC). HDR repair does not take place in G0 CD34+ HSPCs. Representative data from experiments performed with three different mPB donors and n = 2 biological replicates per donor. Mean ± SD shown. ^∗∗^p < 0.01 by unpaired t test. (D) Cell cycle profiles of CD34+ cells 6 days in culture (3 days in SC media and 3 additional days in SC media or XRC). Three days in XRC induces quiescence in 30% of the cells. Representative data from experiments performed with three different mPB donors and n = 2 biological replicates per donor. Mean ± SD shown. ^∗∗^p < 0.01 by unpaired t test. (E) Editing outcomes in CD34+ cells 6 days in culture (3 days in SC media and 3 additional days in SC media or XRC). Three days in XRC results in HDR edits in G0 CD34+ HSPCs. Representative data from experiments performed with three different mPB donors and n = 2 biological replicates per donor. Mean ± SD shown. (F) Percentage of CD34+ cells that are CD34+CD38+ (progenitors) versus CD34+CD38− (EE HSPCs) 6 days in culture. CD34+ cells that regained quiescence in XRC include a higher proportion of EE HSPCs than CD34+ cells maintained solely in SC media. Representative data from experiments performed with three different mPB donors and n = 2 biological replicates per donor. Mean ± SD shown. ^∗∗^p < 0.01 by unpaired t test. See also Figure S4.

We edited CD34+ HSPCs and cultured them in SC media to allow them to enter the cell cycle (Figure 4A). On the day of electroporation, 50% of CD34+ HSPCs were quiescent, as expected (Figure S4A). Two days after editing, we sorted cells based on cell cycle and quantified editing outcomes by NGS. We found that at this time point most cells had exited G0 and were in G1 or S-G2-M (Figure 4B), although less of EE HSPCs were in S-G2-M than the progenitors (Figure S4B). As before, HDR alleles were almost completely absent from the remaining G0 cells but present in G1 and S-G2-M cells, whereas NHEJ alleles were present in all stages of the cell cycle (Figure 4C). HDR/NHEJ ratio was 7 times lower in G0 cells than G1 and S-G2-M cells. We then kept the remaining HSPCs in SC to allow cycling to continue for another 3 days or moved them to XRC to induce quiescence. Six days after editing (3 days in SC and 3 additional days in either SC or XRC), we sorted based on cell cycle and quantified repair outcomes by NGS.

HSPCs that were maintained continuously in SC media (SC SC HSPCs) were almost completely lacking in G0 cells by 6 days after editing (Figure 4D). The remaining cells, which were only in G1 or S-G2-M, harbored high levels of HDR alleles (Figure 4E). In contrast, cells moved to XRC (SC XRC HSPCs) had almost 40% G0 cells and relatively few cells in S-G2-M (Figure 4D). As XRC treatment induces quiescence and SC promotes expansion and differentiation, SC XRC cultures overall yielded ∼50% less cells than SC SC cultures (Figure S4C). Notably, 60% of the primitive EE HSPCs returned to quiescence compared to 30% of CD34+38+ progenitors (Figure S4D). We found that the G0 cells in XRC now harbored high levels of HDR alleles and were in fact comparable in HDR to G1 and S-G2-M cells (Figure 4E). We show that XRC treatment maintains stemness (Figure S4E) and supports viability (Figure S4F) and is distinctive from the omission of cytokines that generally leads to a loss of CD34+ expression and viability (Figures S4E and S4F). We further found that post-treatment with XRC led to enrichment in EE HSPCs compared to continued culture in SC (Figure 4F). However, because using CD34+ CD38− to enrich for engrafting stem cells has its limitations especially under prolonged culture, we further tested the effects of XRC through single-cell RNA sequencing (scRNA-seq) and *in vivo* xenotransplantation.

**Figure 5:**
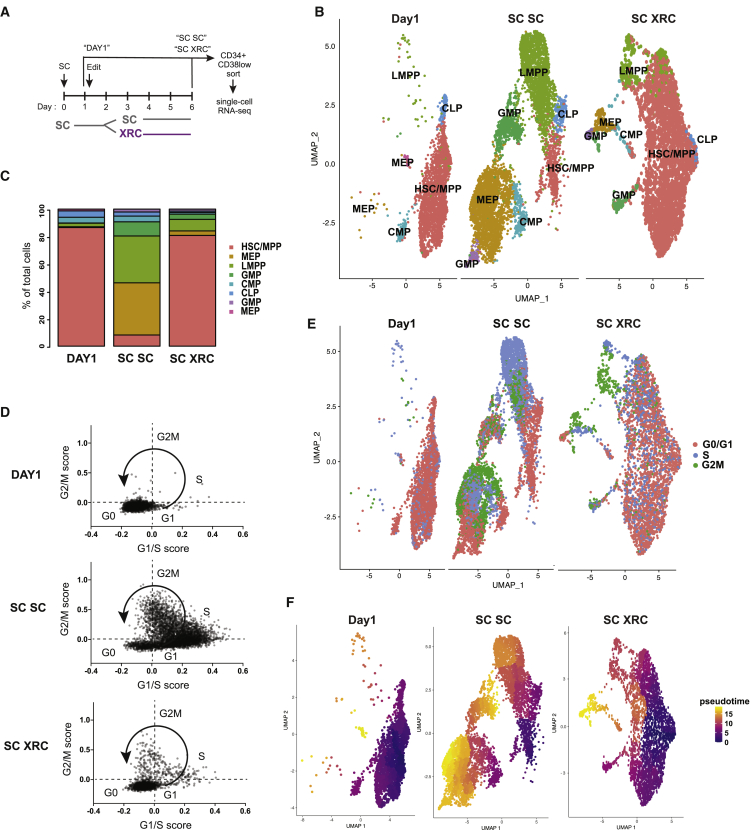
Single-Cell RNA Sequencing Indicates That XRC Treatment Leads to the Maintenance of Quiescent HSC/MPPs (A) Schematic of the workflow for single-cell RNA sequencing (scRNA-seq). CD34+ HSPCs are placed in SC culture for 1 day before editing and cycle for 2 additional days in SC culture, and then quiescence is induced with XRC for 3 days before the cells are subjected to FACS of CD34+CD38low cells and sequenced. (B) UMAPs of scRNA-seq using Seurat 3□s integrated analysis for day 1+SC SC and anchor transfer to SC XRC. Number of cells in day 1 = 2,454, SC SC = 5,686, and SC XRC = 3,595. (C) Fraction of cell assignments for each dataset. (D) Cell cycle status of single cells based on G1/S and G2/M scores. (E) Cell cycle status predicted through gene expression. (F) Pseudotime estimates with HSC/MPPs as the root showing the inferred differentiation of day 1/SC SC and SC XRC datasets. See also Figures S5 and 56 and Table S1.

To thoroughly characterize the effects of re-quiescence on HSPC sub-populations, we carried out scRNA-seq on HSPCs maintained continuously in SC after Cas9 editing (SC SC HSPCs) or moved to XRC briefly after Cas9 editing (SC XRC HSPCs). We also performed scRNA-seq on cells that have only been in SC culture for 1 day, as a control for primitive cells with minimum exposure to *ex vivo* culture. To maximize the resolution of each primitive population, we enriched relatively rare HSCs by sorting CD34+ CD38low HSPCs from CD34+ HSPCs that had been treated with either SC or XRC for 3 days (Figure 5A).

To identify conserved subpopulations of CD34+ CD38low HSPCs in control datasets, we performed integrated analysis of day 1 and SC SC datasets by using the Seurat v3.1.4 package (Butler et al., 2018; Stuart et al., 2019). Alignment and integrated clustering of the day 1 and SC SC datasets revealed seven distinct clusters, which we assigned to HSPC subpopulations by comparison of cluster marker genes to transcriptional signatures of defined cell types in previously published bulk and scRNA-seq datasets (e.g., HLF and AVP for HSC/MPPs, CTSG and IGLL1 for lymphomyeloid-primed progenitors (LMPPs), DNTT and JCHAIN for common lymphoid progenitors (CLPs), CNRIP1 and FCER1A for common myeloid progenitors (CMPs), F13A1, PF4 for granulocyte monocyte progenitors (GMPs), and GATA1 and HBD for megakaryocytic erythroid progenitors [MEPs]) (Figures S5 and S6; Table S1; Buenrostro et al., 2018; Corces et al., 2016; Velten et al., 2017). We identified one cluster each as HSC/MPPs, LMPPs, CLPs, and CMPs and two clusters each as GMPs and MEPs in the day 1/SC SC integrated dataset (Figures 5B, S5, and S6). As expected, extended treatment in SC SC led to differentiation and loss of primitive HSPCs. Specifically, 86.5% of the CD34+ CD38low HSPCs sequenced 1 day after thaw clustered as HSC/MPPs, whereas only 8.1% of SC SC CD34+ CD38low cells cluster as HSC/MPPs (Figures 5B, 5C, S6B, and S6C).

Having identified cell types from the control scRNA-seq datasets, we transferred these labels and clusters to the experimental SC XRC condition and compared the abundances of each cell population (Stuart et al., 2019). Strikingly, SC-XRC-treated CD34+ CD38low cells contained 80.8% of cells with an HSC/MPP transcriptional signature, which is approximately 10 times higher than that of SC SC (Figures 5B and 5C). SC SC 34+38low cells were highly enriched in GMPs, MEPs, LMPPs, and CMPs, whereas SC XRC 34+38low cells were highly enriched in more primitive HSC/MPPs. (Figures 5B and 5C). Transcripts important for HSC regenerative potential were upregulated in the HSC subset under both the day 1 and SC XRC conditions (Figures S5 and S6D), including HLF and PROM1 (CD133) (Hess et al., 2006; Komorowska et al., 2017). Although day 1/SC SC and SC XRC conditions followed a similar trend for the expression of marker genes, they were not completely identical because (1) many genes are expressed at much lower level in the SC XRC dataset due to induction of quiescence by XRC and (2) most cells in the SC XRC condition are assigned to the HSC/MPP cluster, leaving limited numbers of cells in other clusters in the SC XRC condition (Figure S5). Overall, these data indicate that re-quiescence with SC XRC helps maintain an HSC program.

We analyzed the single-cell expression of cell cycle markers to determine whether the XRC re-quiescence strategy increased the proportion of non-cycling cells. Each cell was scored for cell cycle status based on its expression of 43 G1/S markers and 55 G2/M markers (Butler et al., 2018; Kowalczyk et al., 2015; Tirosh et al., 2016; Figures 5D, 5E, and S6E). Most day 1 cells were classified as G0 because they lacked both G1/S markers (e.g., TYMS, PCNA, MCM2, and CDCA7) and G2/M markers (e.g., CDK1, CKS1B, CCNB2, and CDC20). SC SC cells were clearly progressing through the cell cycle, as they expressed very high amounts of G1/S and G2/M markers. In contrast, SC XRC cells were more similar to day 1 cells and mostly scored as G0 (Figure 5D). This includes cells identified as HSC/MPP by single-cell global transcriptome analysis (Figure 5B).

We further analyzed the single-cell transcriptomic data to estimate the differentiation progress of individual HSPCs using pseudotime analysis (Cao et al., 2019; Trapnell et al., 2014). Tracking transcriptome changes as a function of progress along the learned differentiation trajectory, we found that SC SC treatment yields increasingly heterogeneous subpopulations that are mostly advanced in pseudotime and differentiation. In contrast, day 1 and SC-XRC-treated cells mostly consist of primitive populations with a small proportion of differentiated cells (Figure 5F). Overall, our scRNA-seq data reveal that continued culture in SC after Cas9 editing drives cycling and differentiation, whereas moving edited cells to XRC after a brief period of cycling induces quiescence and increases the proportion of transcriptionally defined HSC/MPPs.

**Figure 6:**
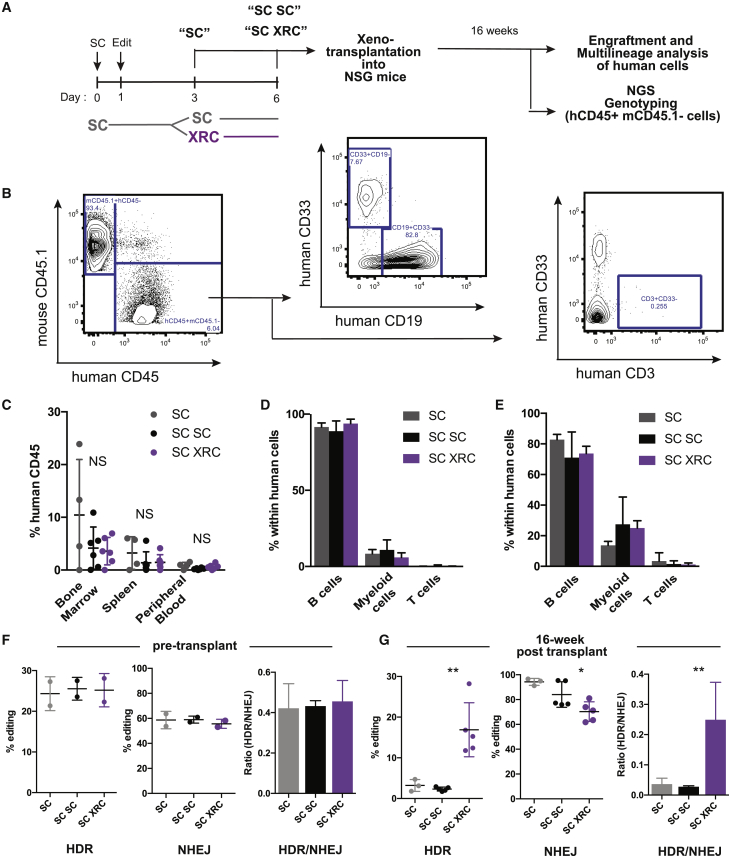
Xenotransplantation Indicates That XRC Treatment after Gene Editing Leads to Efficient HDR in Long-Term Engrafting HSCs (A) Schematic of the workflow for xenotransplantation. CD34+ HSPCs are placed in SC culture for 1 day before editing and cycle for 2 additional days in SC culture, and then quiescence is induced with XRC for 3 days before xenotransplantation into NSG mice. Sixteen weeks post-transplant, bone marrow is collected for engraftment analysis, multilineage analysis, and NGS genotyping; spleen is collected for engraftment analysis; and peripheral blood is collected for engraftment and multilineage analysis. (B) Gating strategy for measuring human cell engraftment and multilineage differentiation. (C) Human cell engraftment (human CD45/mouse CD45.1) 16 weeks after transplant in the bone marrow, spleen, and peripheral blood of NSG mice. Data from individual mice and mean ± SD shown. n = 4 or 6. NS, not significantly different by unpaired t test. (D and E) Percentage of indicated lineages (B cells [CD19], myeloid cells [CD33], and T cells [CD3]) within the human cell graft in the bone marrow (D) and peripheral blood (E) of NSG mice. Data from individual mice and mean ± SD shown. n = 4 or 6. (F and G) Editing outcomes in CD34+ HSPCs before transplanting into NSG mice (F) and in hCD45+mCD45.1− cells from the bone marrow of NSG mice 16 weeks after transplant (G) (SC, 3 days in SC media; SC SC, 6 days in SC media; SC XRC, 3 days in SC media and 3 additional days in XRC before transplant). Data from individual mice and mean ± SD shown. n = 3 or 5 for each condition (mice with engraftment < 2% were excluded from this analysis due to insufficient cell number for amplicon sequencing). ^∗^p < 0.05, ^∗∗^p < 0.01 by unpaired t test. Representative data from experiments performed with two different mPB donors and n = 4–6 biological replicates per donor.

To evaluate the effect of XRC treatment on editing in long-term engrafting HSCs, we transplanted edited SC SC HSPCs and SC XRC HSPCs into immunodeficient non-obese diabetic (NOD) severe combined immunodeficiency (SCID) Il2rg^−/−^ (NSG) mice (Figure 6A). We also xenotransplanted HSPCs that had been cultured in SC for only 3 days as a negative control for the effects of extended culture. Engraftment was measured by the percentage of human CD45 versus mouse CD45.1 (Figure 6B).

We found no significant differences in engraftment of human cells in mice transplanted with CD34+ cells from different culture conditions (bone marrow, SC: 10.425% ± 10.54, SC SC: 2.90% ± 2.27, SC XRC: 3.59% ± 2.59; spleen, SC: 3.23% ± 3.08, SC SC: 1.42% ± 2.08, SC XRC: 1.47% ± 1.44; peripheral blood, SC: 0.81% ± 0.64, SC SC: 0.30% ± 0.27, SC XRC: 0.61% ± 0.49) (Figure 6C). Furthermore, culture condition did not affect the potential for *ex vivo* multilineage differentiation (Figures 6B, 6D, and 6E). Notably, in SC-XRC-treated cells, we found that high levels of HDR alleles persisted after long-term *in vivo* engraftment. Most HDR alleles were instead lost under the SC and SC SC conditions (Figures 6F and 6G). SC XRC HDR was 5- to 6-fold higher than either the SC or SC SC conditions, and we found levels of HDR in SC-XRC-treated long-term engrafting human cells as high as 28% (Figure 6G). NHEJ alleles were proportionately reduced after SC XRC treatment, suggesting a tradeoff between HDR and NHEJ within equivalent total editing. The HDR/NHEJ ratio after SC XRC treatment was approximately 0.25 (Figure 6G). Together with our *ex vivo* data, these findings indicate that XRC enriches for HDR in repopulating stem cells by encouraging HDR-edited cells to re-enter G0 (Figure 6G). In summary, we have developed a strategy to enable high-efficiency HDR in primitive and quiescent HSPCs. This strategy allows HSPCs to briefly cycle after Cas9-mediated induction of a DSB to allow HDR and then places cells back into quiescence after HDR alleles have been acquired.

## Discussion

Our data provide an approach to “scarlessly” (without selectable markers) introduce mutations to human HSCs for fundamental research, to suggest ways to treat gene-edited HSCs for therapeutic purposes, and to also shed light on fundamental HSC biology. The DNA repair decisions after a DSB in primitive human hematopoietic cells are essential for cell survival; yet, they are underexplored due to difficulties in studying human HSCs. Aged human hematopoietic cells show elevated levels of unresolved DSBs and increased mutation frequencies, but the mechanisms underlying DSB repair in these cell types are largely unknown (Beerman et al., 2014; Genovese et al., 2014a; Rossi et al., 2007; Rübe et al., 2011; Cancer Genome Atlas Research Network et al., 2013). Here, we have used CRISPR genome editing to induce a precise DSB in mixed CD34+ HSPCs and measured its repair in various cell subtypes and phases of the cell cycle by a combination of FACS and NGS of sorted populations. This general experimental strategy could broadly accelerate in-depth probing of DNA repair decisions in many different cell types with available immunophenotypic markers.

We found that genome editing of CD34+ HSPCs leads to high levels of NHEJ in multiple cell subtypes but that HDR is preferentially missing from more primitive quiescent cells. Instead, HDR accumulates in relatively differentiated cells and immunophenotypically primitive cells that have exited quiescence. Several groups have reported that genome editing CD34+ HSPCs leads to high-efficiency HDR in relatively short-term *in vitro* culture that drops dramatically during long-term *in vivo* engraftment (Dever et al., 2016; DeWitt et al., 2016; Genovese et al., 2014b; Hoban et al., 2015; Wang et al., 2015; Wu et al., 2019). This is true even with very different modalities of Cas9 (mRNA, recombinant protein), guide RNA (synthetic, adeno-associated virus (AAV) expressed), and HDR donor (single-stranded DNA, AAV6) (Dever et al., 2016; DeWitt et al., 2016; Genovese et al., 2014b; Hoban et al., 2015; Kim et al., 2014; Wang et al., 2015). A recent report also found that a specific subtype of base editing by nucleotide deaminases at the BCL11A erythroid enhancer is less efficient in quiescent human HSCs than in non-quiescent progenitor cells (Zeng et al., 2020). Our results indicate that the observed *in vivo* lack of HDR is not caused by an inability to target immunophenotypic LT-HSCs, nor toxicity caused by the act of performing HDR itself (Ihry et al., 2018), but is instead because the repopulating HSCs are in an inappropriate phase of the cell cycle to perform HDR. Forcing cycling HSCs into quiescence immediately after editing is sufficient to completely abrogate HDR alleles, and allowing HSCs to cycle briefly and then inducing quiescence enables HDR.

Mechanistic investigations of DNA repair have established that HDR is preferentially active in the S/G2 stages of the cell cycle, probably to avoid deleterious telomere fusions that can occur if HDR is active during mitosis (Orthwein et al., 2014). Non-mitotic cells such as HSCs, therefore, represent a challenge. For HSCs, division is central to self-renewal and differentiation, but stemness is intricately linked to long-term quiescence (Ema et al., 2000; Morrison and Weissman, 1994; Suda et al., 1983). Prolonged *in vitro* culture of HSCs leads to a loss of stemness, entry into the cell cycle, and poor engraftment (Morrison and Kimble, 2006; Wilson et al., 2008). Therefore, there is a tension between HDR editing and the maintenance of stemness by quiescence. LT-HSCs may need to lose a defining feature of their stemness to obtain HDR edits.

One might avoid HDR entirely and instead pursue NHEJ-based editing. In HSCs, this approach shows promise for the treatment of SCD, in which disruption of various repressor elements leads to re-expression of protective fetal hemoglobin (Bauer et al., 2013; Bjurström et al., 2016; Canver et al., 2015; Chang et al., 2017). NHEJ is well-represented in long-term engrafting HSCs during genome editing (Dever et al., 2016; DeWitt et al., 2016; Genovese et al., 2014b; Hoban et al., 2015; Wang et al., 2015; Wu et al., 2019), and here, we show by immunophenotyping and cell cycle analysis that HSCs in G0 are fully capable of accumulating NHEJ alleles. However, limiting oneself to NHEJ-based editing does not fully tap the potential of genome editing. Many fundamental questions are best answered by surgically replacing genomic sequences, and relatively few genetic diseases can be cured by NHEJ-based sequence disruption.

Our lab and others have found that the drop in levels of HDR after long-term CD34+ engraftment is reflected in low HDR but high NHEJ in the quiescent LT-HSC subpopulation. In contrast, cycling progenitor cells and even MPPs exhibit significant levels of HDR. Previous efforts to increase the HDR/NHEJ ratio in LT-HSCs focused on use of a Cas9-geminin fusion mRNA to reduce nuclease activity in G1, where NHEJ is prevalent but HDR is low. This procedure reduces deleterious NHEJ alleles but does not increase absolute levels of HDR (Lomova et al., 2018). We pursued a complementary approach, reasoning that rapid RNP-based editing followed by progression through at least one cell cycle and subsequent re-quiescence should increase HDR alleles in HSCs (Branzei and Foiani, 2008; Hustedt and Durocher, 2016). SC XRC treatment indeed resulted in quiescent LT-HSCs that exhibit 5- to 6-fold increases in HDR up to almost 30% of alleles, which is close to those observed in cycling progenitors. Our data show that treatment with rapamycin and CHIR99021 triggers pathways that affect cell cycle control and lead to a change in preference between NHEJ and HDR. In principle, the SC XRC strategy could be combined with Cas9-geminin to simultaneously reduce NHEJ and increase HDR in HSCs because NHEJ is still higher than HDR with our strategy, which leads to a high percentage of cells with null alleles. But, further optimization to establish timing with which the nuclease mRNA is introduced relative to re-quiescence would be required before the combined strategy can be used clinically.

By integrating multiple scRNA-seq datasets, we used transcriptomics to identify changes in HSPC subpopulations and their cell cycle status in response to re-quiescence. We found that XRC treatment after Cas9 editing results in most cells with transcriptional profiles of quiescent HSCs, similar to CD34+CD38low HSPCs immediately after thawing. Although mouse HSPCs have been extensively studied using scRNA-seq, there are relatively few studies analyzing human HSPCs (Buenrostro et al., 2018; Kowalczyk et al., 2015; Månsson et al., 2007; Moignard et al., 2013; Pellin et al., 2019; Povinelli et al., 2018; Velten et al., 2017; Wilson et al., 2015). Combining single-cell transcriptomics with single-cell genotyping would be a great next step to further uncover the relationship between gene editing and cell identity in human HSPCs under XRC treatment and beyond.

Directly addressing the tension between quiescence and HDR is critical to fully achieve the potential of genome editing. Multiple types of primary cells potentially suffer from poor HDR that may be linked to quiescence (Bressan et al., 2017; Schwank et al., 2013; Urnov et al., 2010; Zhu et al., 2017). Notably, genome editing of primary human T cells requires activation via anti-CD3/anti-CD28 stimulation to achieve efficient editing. The re-quiescence strategy we develop here could be applicable beyond HSCs, although one barrier is the paucity of culture models for various types of primary and stem cells. The potential toxicities of mTOR and GSK inhibitors will also need to be thoroughly tested before they are considered for therapeutic purposes. However, our data indicate that culture conditions and a target cell’s underlying biology can be just as important as editing modality to achieve desired genomic outcomes.

## STAR★Methods

### Key Resources Table

**Table:** 

REAGENT or RESOURCE	SOURCE	IDENTIFIER
**Antibodies**		

Percp-Cy5.5 Mouse Anti-Human CD34 (Clone 561)	Biolegend	343612; RRID: AB_2566788
PE-Cy7 Mouse Anti-Human CD38 (Clone HB7)	BD Biosciences	335790; RRID: AB_399969
PE Mouse Anti-Human CD90 (Clone 5E10)	BD Biosciences	555596; RRID: AB_395970
FITC Mouse Anti-Human CD45RA (Clone HI100)	BD Biosciences	555488; RRID: AB_395879
BV421 Rat Anti-Human CD49f (Clone GoH3)	BD Biosciences	562598; RRID: AB_2737673
Pacific Blue anti-Human CD45 (Clone HI30)	Biolegend	304029; RRID: AB_2174123
FITC anti-mouse CD45.1 Antibody (Clone A20)	Biolegend	110706; RRID: AB_313495
APC anti-mouse TER-119/Erythroid Cells Antibody (Ly-76)	Biolegend	116212; RRID: AB_313713
PE Mouse Anti-Human CD19 (HIB19)	BD Biosciences	555413; RRID: AB_395813
APC Mouse Anti-Human CD33 (WM53)	BD Biosciences	551378; RRID: AB_398502

**Chemicals, Peptides, and Recombinant Proteins**		

Rapamycin	EMD Millipore	553210
CHIR99021	EMD Millipore	361559
Retinoic Acid (ATRA)	Sigma	R2625
StemSpan CC110	StemCell Technologies, Inc.	2697
Cas9-NLS	UC Berkeley	NA
Hoechst33342	ThermoFisher	H3570
Pyronin Y	Biotang	BTBB602
Primestar GXL DNA Polymerase	Takara Biosciences	R050A
Fixable viability stain 660	BD Biosciences	564405

**Critical Commercial Assays**		

FITC mouse anti-Ki67 kit	BD Biosciences	556026
FITC Annexin V Apoptosis Detection Kit with PI	Biolegend	640914
P3 primary cell 96-well nucleofector kit	Lonza	V4SP-3096
Illumina MiSeq reagent kit v2 (300-cycles)	Illumina	MS-102-2002
Illumina HiSeq 3000/4000 SBS kit (300 cycles)	Illumina	FC-410-1003
10X Single-cell 3′ library gel bead kit v2	10Xgenomics	PN-120267

**Deposited Data**		

Amplicon sequencing and single-cell RNA-sequencing datasets	This paper	BioProject ID PRJNA498122

**Experimental Models**		

Mobilized peripheral blood CD34+ Stem/Progenitor cells	AllCells	mPB015F
NOD.Cg-*Prkdc*^*scid*^*Il2rg*^*tm1Wjl*^/SzJ (NSG) mouse	Charles River	005557

**Oligonucleotides**		

Synthetic sgRNA targeting the HBB locus	Trilink	NA
FP1: cacttagacctcaccctgtg	IDT	NA
FP2: tatgggacgcttgatgttttct	IDT	NA
RP1: tatgggacgcttgatgttttct	IDT	NA
RP2: ctctgcctattggtctattttccca	IDT	NA
HBB ssODN: TCAGGGCAGAGCCATCTATTG CTTACATTTGCTTCTGACACAACTGTGTTCA CTAGCAACCTCAAACAGACACCATGGTGCA CCTGACTCCTGTAGAGAAGTCTGCGGTTACT GCCCTGTGGGGCAAGGTGAACGTGGATGAA GTTGGTGGTGAGGCCCTGGGCAGGT	IDT	NA

**Software and Algorithms**		

CRISPResso	Pinello et al., 2016	https://github.com/lucapinello/CRISPResso
Seurat v3.1.4	Butler et al., 2018; Stuart et al., 2019	https://satijalab.org/seurat/
Monocle3 v0.2.1	Trapnell et al., 2014	http://cole-trapnell-lab.github.io/monocle-release/monocle3/
R v3.6.3	R Development Core Team	https://www.r-project.org/

**Other**		

SFEMII	StemCell Technologies, Inc.	09655
X-VIVO15	Fisher	BW04744Q

### Resource Availability

#### Lead Contact

Further information and requests for reagents may be directed to, and will be fulfilled by, the Lead Contact, Jacob Corn (jacob.corn@biol.ethz.ch).

#### Materials Availability

This study did not generate new unique reagents.

#### Data and Code Availability

The datasets generated during this study are available at BioProject ID PRJNA498122.

### Experimental Model and Subject Details

Cryopreserved wild-type human mobilized peripheral blood CD34+ HSPCs from multiple volunteer donors including male and female whose age ranged from 20-35 were purchased from Allcells, Inc.

### Method Details

#### Primary Cell Culture

CD34+ HSPCs were cultured in SC (SFEMII + CC110 (StemCell Technologies)) media, XRC (X-VIVO15 (Lonza) + 5nM Rapamycin (EMD Millipore) + 3uM CHIR99021 (EMD Millipore)), or SC + 5uM All-trans retinoic acid (Sigma) media unless otherwise noted.

#### Electroporation for editing experiments

Cas9 RNP synthesis was carried out as previously described (DeWitt et al., 2016). Briefly, 75pmol of Cas9-NLS (UC Berkeley, Berkeley, CA) was mixed slowly into Cas9 buffer (20mM HEPES (pH 7.5), 150mM KCl, 1mM MgCl_2_, 10% glycerol and 1mM TCEP) containing 75pmol of synthetic sgRNA targeting the HBB locus (Synthego). The resulting 7.5ul mixture was incubated for 15minutes to allow RNP formation. 2x10^−5^ CD34+ HSPCs were harvested, washed once with PBS, and resuspended in 20ul of P3 nucleofection buffer (Lonza, Basel, Switzerland). 7.5ul of RNP mixture and 20ul of cell suspension were combined and added into a Lonza 4d strip nucleocuvette and were electroporated with program ER-100. 200ul pre-warmed media was added to each nucleocuvette and electroporated cells were transferred to culture dishes. Editing outcomes were measured 1-5 days post-electroporation by Next Generation Amplicon Sequencing.

#### PCR and Next-Generation Amplicon Sequencing preparation

50-100ng of genomic DNA from edited CD34+ cells was amplified at HBB sites using primer set 1 (Figure S1A). The PCR products were SPRI cleaned, followed by amplification of 20-50ng of the first PCR product in a second 12 cycle PCR using primer set 2 (Figure S1A). Then the second PCR products were SPRI cleaned, followed by amplification of 20-50ng of the second PCR product in a third 9 cycle PCR using illlumina compatible primers (primers designed and purchased through the Vincent J. Coates Genomics Sequencing Laboratory (GSL) at University of California, Berkeley), generating indexed amplicons of an appropriate length for NGS. Libraries from 100-500 pools of edited cells were pooled and submitted to the GSL for paired-end 300 cycle processing using a version 3 Illumina MiSeq sequencing kit (Illumina Inc., San Diego, CA) after quantitative PCR measurement to determine molarity.

#### Next-Generation Amplicon Sequencing analysis

Samples were deep sequenced on an Illumina MiSeq at 300bp paired-end reads to a depth of at least 10,000 reads. A modified version of CRISPResso (Pinello et al., 2016) was used to analyze editing outcomes. Briefly, reads were adaptor trimmed then joined before performing a global alignment between reads and the reference and donor sequences using NEEDLE (Li et al., 2015). Rates of HDR are calculated as total reads that successfully convert the main (non-PAM out) edit site and have no insertions or deletions within three basepairs to each side of the cutsite divided by the total number of reads. NHEJ rates are calculated as any reads where an insertion or deletion overlaps the cutsite or occurs within three basepairs of either side of the cutsite divided by the total number of reads.

#### Immunofluorescence

##### Immunophenotypic analysis assays

Human CD34+ cells with or without editing were were first stained with fixable viability stain 660 (1:1000, BD) for 5 min in 37**°**C and then were stained with Percp-Cy5.5-anti-CD34 (1:50), PE-Cy7-anti-CD38 (1:50), PE-anti-CD90 (1:30), FITC-anti-CD45RA (1:25), and BV421-anti-CD49f (1:30) (all of the antibodies are from BD) for 30 min in 4**°**C. Samples were then sorted on Aria Fusion Cell Sorter (BD) or analyzed on LSR Fortessa cytometer (BD).

##### Cell cycle analysis assays

For Ki67-Hoechst assays, CD34+ cells with or without editing were first stained with fixable viability stain 660 (1:1000, BD) for 5 min in 37**°**C and were fixed with Cytofix/Cytoperm buffer (BD) for 15 min in 4**°**C. Cells were stained with FITC-anti-KI67 (1:25, BD, 556027) for 2hours-overnight in Permwash buffer (BD), then with Hoechst 33342 (1:5000; Life Technologies) for 5 min at RT. Samples were sorted on a Aria Fusion Cell Sorter (BD) or analyzed on a LSR Fortessa cytometer (BD). For assessment of immunophenotypic markers together with cell cycle analysis, human CD34+ cells with or without editing were stained with Percp-Cy5.5-anti-CD34 (1:50) and PE-Cy7-anti-CD38 (1:50) for 30 min in 4**°**C before they were stained with fixable viability stain and fixed. For assessment of cell cycle status without fixation (live cell cycle status), cells with or without editing were stained with Hoechst 33342 (1:1000, Invitrogen) for 45min in 37**°**C, and then were stained with Pyronin Y (1:20,000, Invitrogen) for additional 15 min in 37**°**C or were just stained with Pyronin Y for 15 min in 37**°**C. Samples were then sorted on Aria Fusion Cell Sorter (BD) or analyzed on LSR Fortessa cytometer (BD).

##### Apoptosis analysis assays (Annexin V, PI)

Human CD34+ cells with or without editing were first stained with Percp-Cy5.5-anti-CD34 (1:50) and PE-Cy7-anti-CD38 (1:50) for 30 min in 4**°**C before they were washed twice with BioLegend’s Cell Staining Buffer (Biolegend) and stained with FITC Annexin V (Biolegend, 1:20) and PI (Biolegend, 1:10) for 15 minutes at room temperature. Then 400ul of Annexin V binding buffer was added and the samples were analyzed by LSR Fortessa cytometer (BD).

#### Single-cell RNA-sequencing

CD34+ CD38low cells were sorted on Aria Fusion Cell Sorter (BD) and single-cell RNA libraries were prepped using Chromium single cell 3′ reagent kit (10x Genomics) according to protocol, starting with ∼10,000 CD34+ CD38low cells. Prepped libraries were sequenced on an Illumina Hiseq. 10x sequencing data were processed with Cell Ranger v3.0.2 using default parameters and GRCh38 as reference. The three datasets (Day1, SC SC and SC XRC) were further processed using Seurat v3.1.4. Cells with > 200 detected genes and with < 6% of total expression attributed to mitochondrial genes were used for further analysis. Cell cycle phases were scored using Seurat and the difference (S-G2M), together with the percent of mitochondrial expression were used to scale datasets. Cells from Day1 and SC SC were merged using integration anchors, clusters identified (dims = 30, resolution = 0.3) and cluster labels transferred to SC XRC. Cluster identities were assigned using 1) markers employed in other scRNA-seq papers (Buenrostro et al., 2018; Corces et al., 2016; Velten et al., 2017) and 2) markers established in published bulk FACS-sorted RNA-seq data (Table S1). We minimized bias by assigning the cluster identities to Day1 and SC SC datasets first before transferring those identities to the experimental SC XRC dataset. Monocle 3 v0.2.1 with default options and Seurat UMAPs were used to calculate pseudotime. Two partitions were identified for each dataset (Day1+SC SC and SC XRC) with the HSC/MPP and CMP clusters locating on different partitions. The trajectory end points in the HSC/MPP and CMP clusters were chosen as root in each partition and the pseudotime of a manually chosen closest cell between both partitions was added to the second partition.

#### Xenotransplantation and analysis

8- to 12-week-old NOD-SCID-*Il2rg*^*−/−*^ (NSG) mice were purchased by Charles River. At day 3 or 6 of culture, 1 × 10^6^ gene-targeted mobilized peripheral blood-derived CD34+ cells were injected via tail-vein after sub-lethal irradiation (180cGy, X-ray irradiation with RS-2000 irradiator, Rad Source). Bone marrow was harvested 16 weeks after transplant for next-generation amplicon sequencing (NGS genotyping) and multilineage differentiation analysis of human CD45+ cells. For genotyping, bone marrow cells were stained with Pacific Blue-anti-human CD45 (1:50), FITC-anti-mouse CD45.1(1:100), APC-anti-mouse Ter119 (1:100) for 30 min in 4**°**C and were sorted on Aria Fusion Cell Sorter (BD). For multilineage differentiation analysis of human CD45+ cells, bone marrow cells were stained with APC-anti-human CD45 (1:50), AmCyan-anti-mouse CD45.1(1:100), PE-anti-human CD33 (1:100), FITC-anti-human CD19 (1:100), APC-Cy7-anti-human CD3 (1:100) for 30 min in 4**°**C and were analyzed on LSRFortessa (BD).

### Quantification and Statistical Analysis

Statistical analyses were performed with GraphPad Prism (version 7.00 for Mac, GraphPad Software) using unpaired two-tailed t test analysis. Representative data from n ≥ 2 independent experiments are shown in the figures unless otherwise stated. Each experiment included n ≥ 2 biological replicates unless otherwise noted. More detailed information of experimental replicates is given in the figure legends of the corresponding experiments.

All values are given in the text as mean (±SD) and a p value < 0.05 was accepted as significant in all analyses, unless otherwise stated.

## Supplemental Information

Document S1. Figures S1–S6

Table S1. Top 20 Statistically Significant Genes Differentially Expressed among Each Cluster, Related to Figure 5 Cluster identity – Cluster 0: HSC/MPP, Cluster 1: MEP, Cluster 2: LMPP, Cluster 3: GMP, Cluster 4: CMP, Cluster 5: CLP, Cluster 6: GMP, Cluster 7: MEP

Document S2. Article plus Supplemental Information
