# Supplementary material for: Controlled Cycling and Quiescence Enables Efficient HDR in Engraftment-Enriched Adult Hematopoietic Stem and Progenitor Cells
Source: Cell Rep. 2020 Sep 1;32(9):108093. doi: 10.1016/j.celrep.2020.108093 (PMC7487781; doi:10.1016/j.celrep.2020.108093)
Supplement: Document S1. Figures S1–S6 [file mmc1.pdf]

Cell Reports, Volume *32*

Supplemental Information

Controlled Cycling and Quiescence Enables Efficient HDR in Engraftment-Enriched Adult Hematopoietic Stem and Progenitor Cells

Jiyung J. Shin, Markus S. Schröder, Francisco Caiado, Stacia K. Wyman, Nicolas L. Bray, Matteo Bordi, Mark A. Dewitt, Jonathan T. Vu, Won-Tae Kim, Dirk Hockemeyer, Markus G. Manz, and Jacob E. Corn

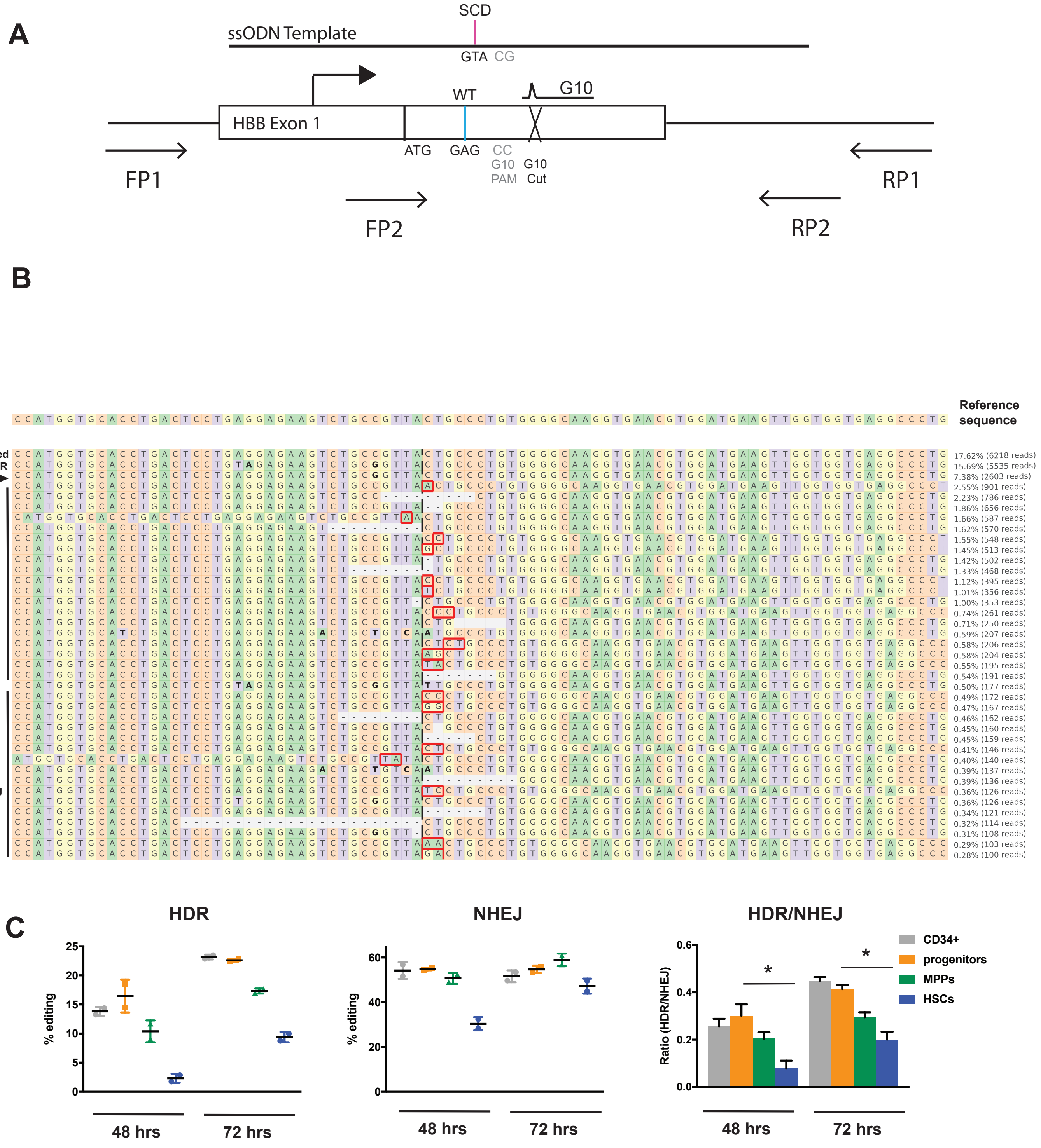

Figure S1. Related to Figure 1: Targeted gene editing at the HBB locus

(A) Schematic of the ssODN template, sgRNA (G10) designed to modify the causative hemoglobin beta (HBB) mutation involved in sickle cell diseasease (SCD), and PCR primers used for amplicon NGS library preparation to assess HDR and NHEJ efficiency. (FP1: tcacttagacctcaccctgtg, RP1: tatgggacgcttgatgttttct, FP2: tatgggacgcttgatgttttct, RP2: ctctgcctattggtc-tattttccca)

(B) Example of an editing result from the amplicon NGS pipeline.

(C) HBB target editing efficiency in CD34+ subpopulations after 48 and 72 hours of electroporation. Representative data from experiments performed with three different mobilized peripheral blood donors and n=3 biological replicates per donor. Mean ± SD shown. **: $p<0.05$ by unpaired t-test.

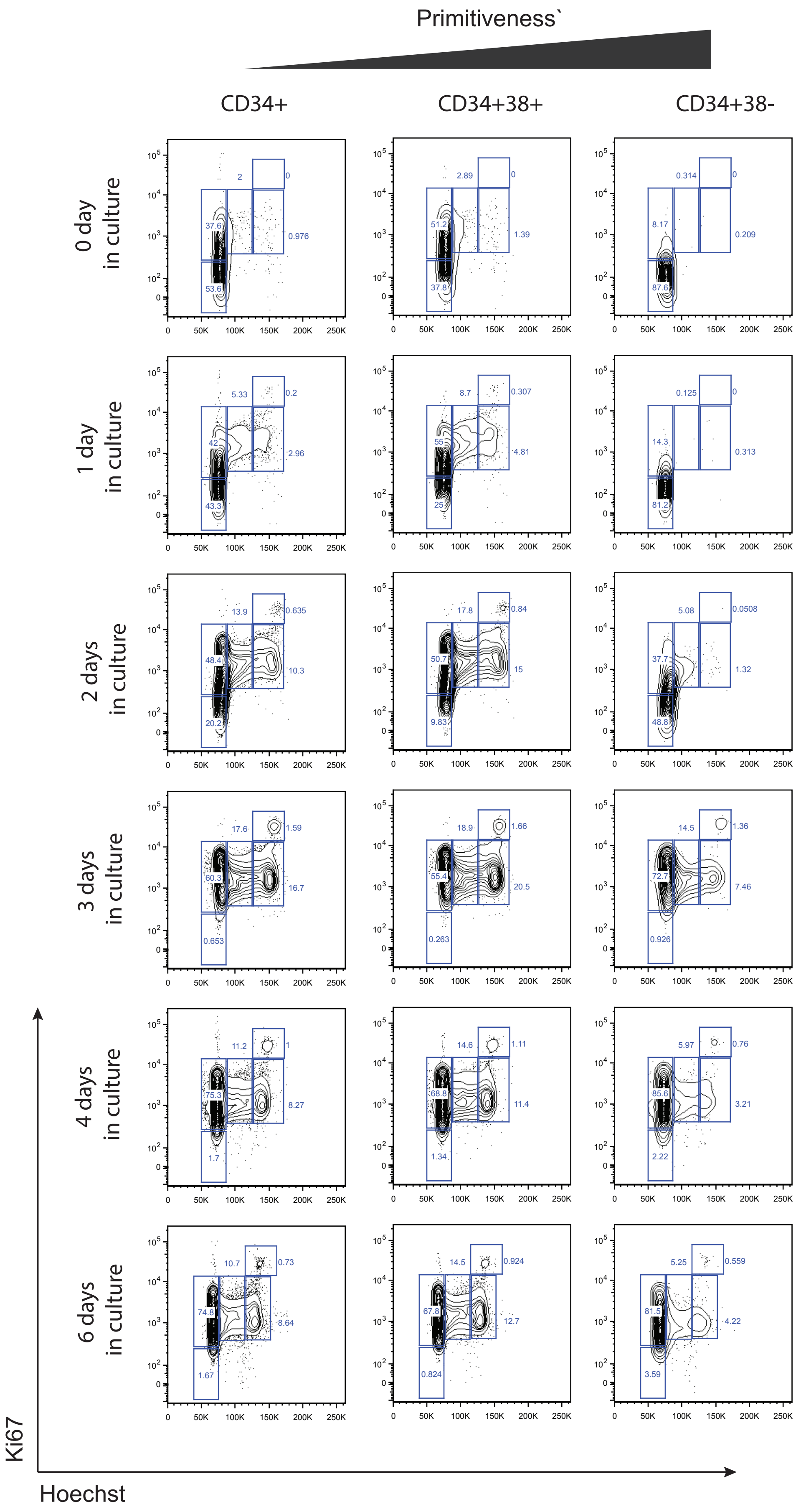

Figure S2. Related to Figure 2: Cell cycle progression of human mPB CD34+ HSPCs in ex vivo culture.
Percentage of G0, G1, S, G2, and M cells in CD34+, CD34+ CD38+ progenitors, and CD34+ CD38- engraftment-enriched (EE) HSPCs after 0-6 days in ex vivo SC culture.

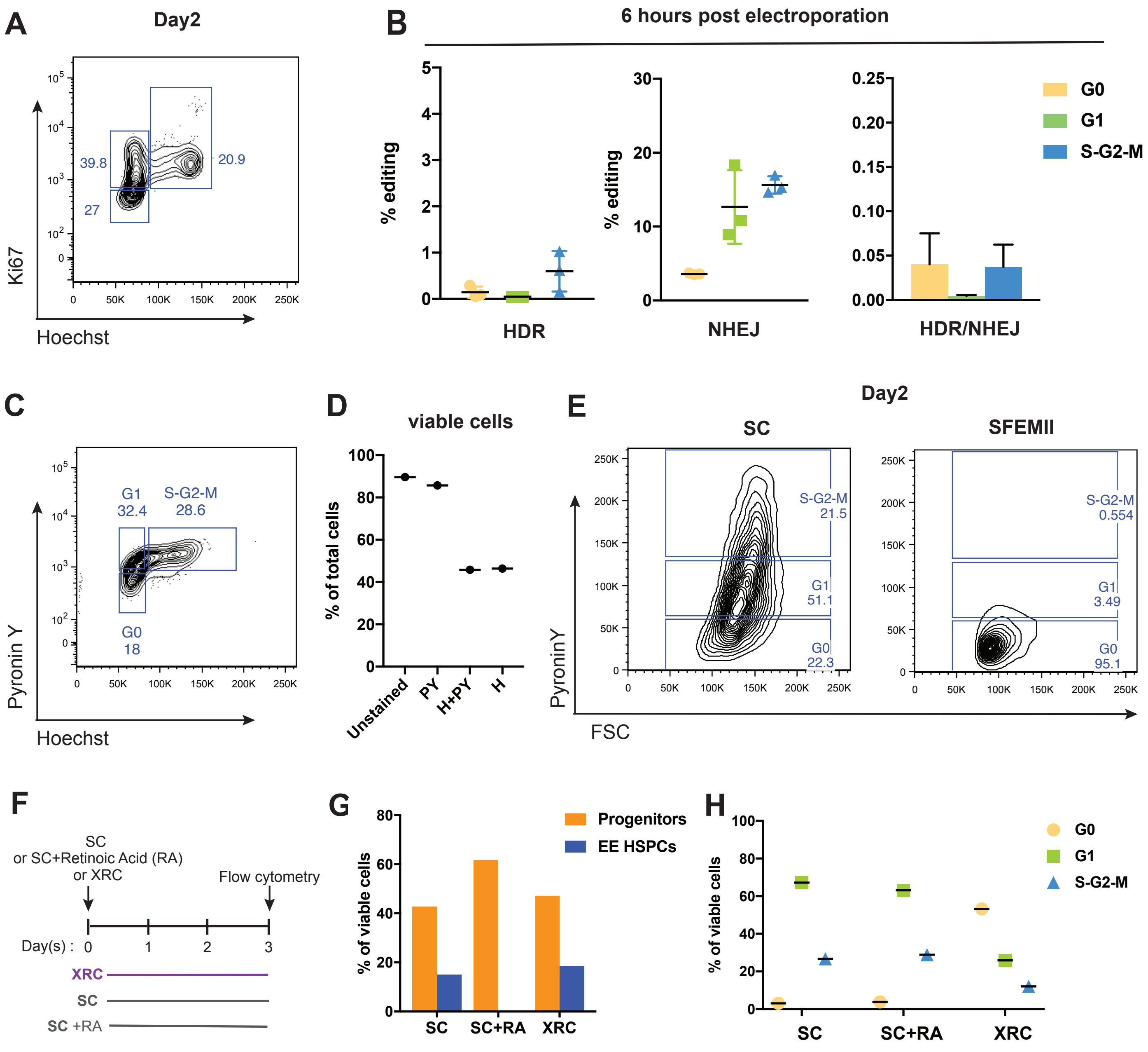

Figure S3. Related to Figure 3: Assessing editing efficiency in CD34+ HSPCs that are in different cell cycle status and establishing an ex vivo culture protocol that maintains quiescence and stemness of CD34+ HSPCs.
(A) Representative flow plot for measuring cell cycle status in Figure 3A.
(B) Editing outcomes in CD34+ population in different cell cycle status 6 hours after electroporation.
Mean ± SD from n=3 biological replicates are shown.
(C) Representative flow plot for live cell cycle measurement.
(D) Viability 2 days after staining with Pyronin Y, Hoechst 33342, or both. Results are representative of two biological replicates.
(E) Representative flow plot for measuring live cell cycle status using Pyronin Y in Figure 3B.
(F) Schematic for the testing of ex vivo culture protocol (SC, SC + Retinoic Acid, XRC) to maintain quiescence without the loss of stemness measured by % of EE HSPCs.
(G) Percentage of progenitors (CD34+ CD38+) vs. EE HSPCs (CD34+ CD38-) among viable cells. Results are representative of two biological replicates.
(H) Percentage of CD34+ HSPCs in G0, G1, and S-G2-M among viable cells. Results are representative of two biological replicates.

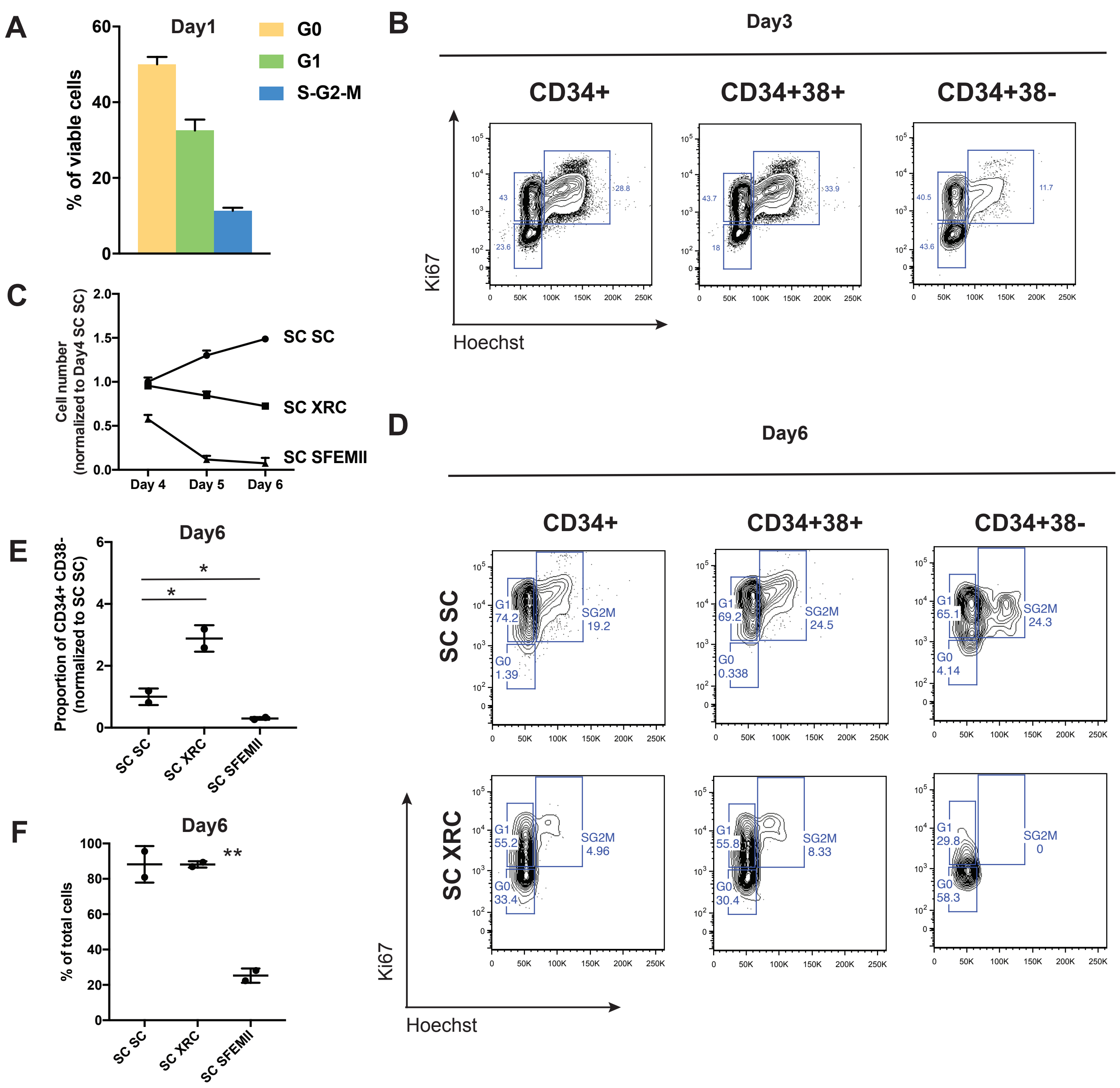

Figure S4. Related to Figure 4: Inducing quiescence via XRC treatment after a short period of cycling yields quiescent, primitive HSPCs

(A) Cell cycle profiles of CD34+ HSPCs at the time of nucleofection (Day1) in Figure 4A.

(B) Representative flow plots for cell cycle status 2 days post electroporation of CD34+, CD34+CD38+ (progenitors), and CD34+ CD38- (EE HSPCs).

(C) Cell number from day4-6 in SC SC, SC XRC, and SC SFEMII normalized to day4 SC SC. Data shown as mean ± SD of 2 biological replicates.

(D) Representative flow plots for cell cycle status 5 days post electroporation of CD34+, CD34+CD38+ (progenitors), and CD34+ CD38- (EE HSPCs).

(E) Percentages of EE HSPCs in SC SC, SC XRC, and SC SFEMII normalized to SC SC. Data shown as mean ± SD of 2 biological replicates. *:p<0.05 by unpaired t-test.

(F) Percentages of viable cells in SC SC, SC XRC, and SC SFEMII. Data shown as mean ± SD of 2 biological replicates. **: p<0.01 by unpaired t-test.

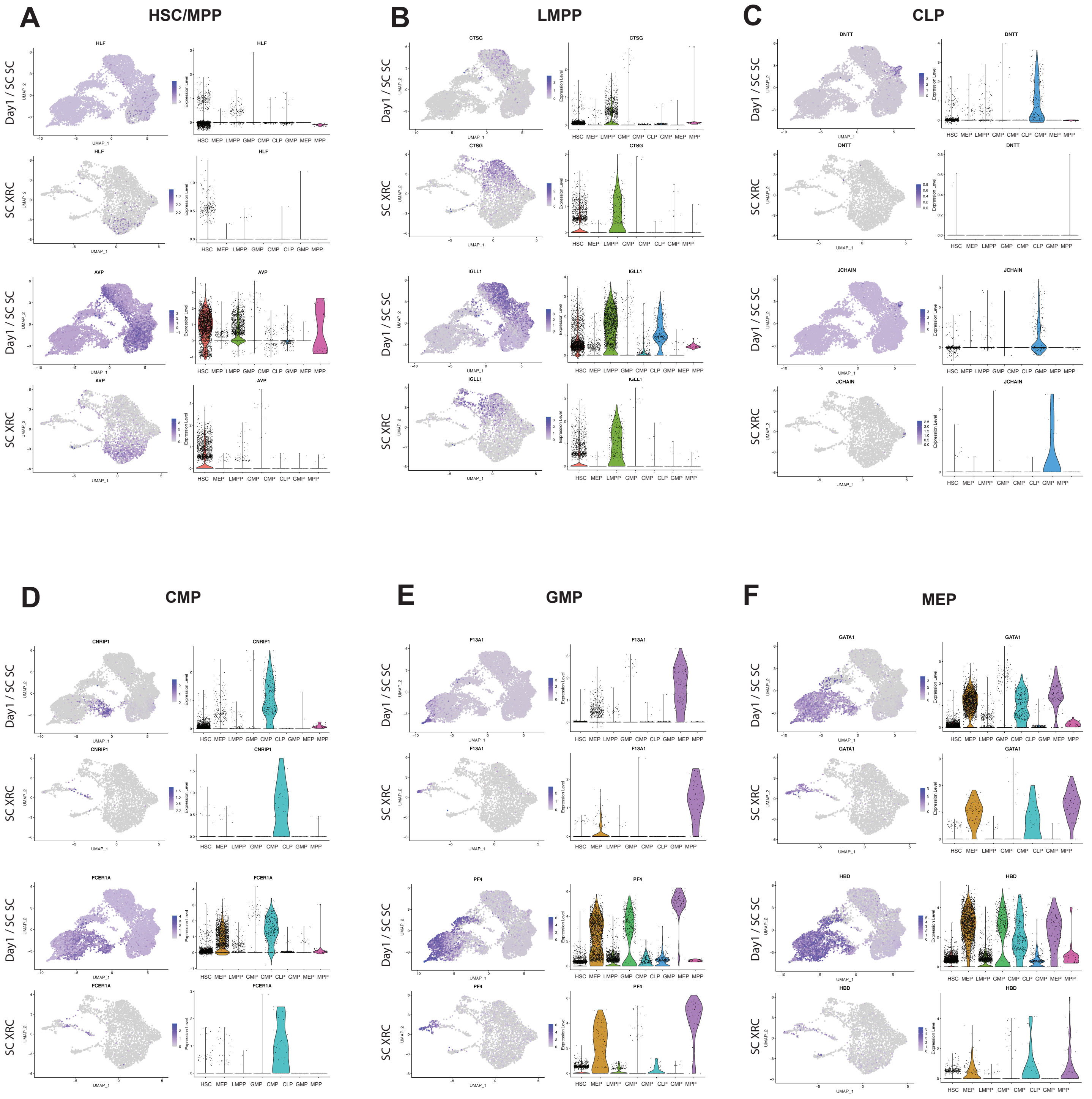

Figure S5. Related to Figure 5: Single cell RNA sequencing indicates that XRC treatment leads to maintenance of quiescent HSC/MPPs

Marker gene expression in Day1/ SC SC, SC XRC dataset. (A) HLF,AVP : HSC/MPP, (B) CTSG, IGLL1: LMPP, (C) DNTT, JCHAIN: CLP, (D) CNRIP1, FCER1A - CMP, (E) F13A1, PF4 : GMP, (F) GATA1, HBD : MEP

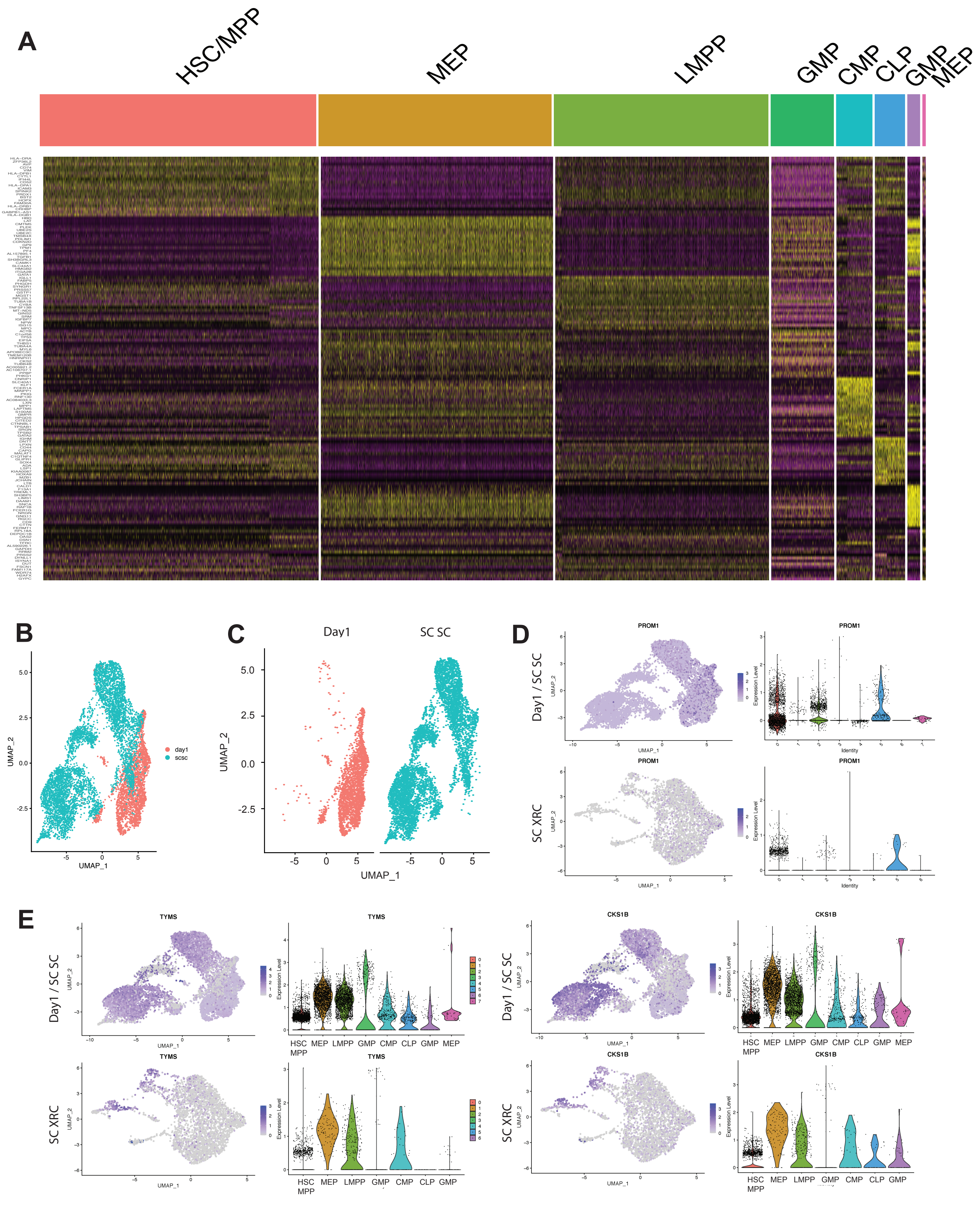

Figure S6. Related to Figure 5: Single cell RNA sequencing indicates that XRC treatment leads to maintenance of quiescent HSC/MPPs

(A) Heatmap showing expression of top 20 statistically significant genes differentially expressed among each cluster. (B) Day1/SC SC integrated UMAP plot. (C) Day1/SC SC integrated UMAP plot separated. (D) Prominin 1 (CD133) gene expression in Day1/ SC SC, SC XRC dataset. (E) Cell cycle marker gene expression in Day1/ SC SC, SC XRC dataset. TYMS: G1/S, CKS1B: G2/M.
